# MIB-1 index predicts low recurrence risk after subtotal resection of meningiomas: a retrospective study of 505 patients

**DOI:** 10.1007/s10143-026-04345-3

**Published:** 2026-06-11

**Authors:** Joonas Laajava, Jenni Määttä, Miikka Korja

**Affiliations:** Department of Neurosurgery, University of Helsinki and Helsinki University Hospital, P.O. Box 266, Helsinki, FI-00029 Finland

**Keywords:** Meningioma, MIB-1 labeling index, Recurrence, Resection, Neural crest, Reoperation

## Abstract

The Ki-67/MIB-1 index, a biomarker of cellular proliferation, holds prognostic value. In meningiomas, MIB-1 correlates with recurrence, with a proposed 4.1% cutoff. This study aimed to identify optimal patient groups for the MIB-1 proliferation index threshold, hypothesizing improved recurrence prediction within stratified subgroups. We performed a retrospective cohort study of adult patients (≥ 18 years) undergoing primary meningioma resection (2005–2018) with available MIB-1 proliferation index and at least five years of MRI follow-up. Meningiomas of all WHO grades were included. Of 505 patients (median age 59 years) meeting inclusion criteria, 340 (67.3%) were female, with meningiomas predominantly WHO grade I (55.8%) or II (41.6%). subtotally resected (STR) WHO grade I meningiomas account for a total of 58 (20.6%) cases out of all 282 (GTR + STR) surgically treated grade I meningiomas. Of these 58 STR grade I meningiomas, 21 (36%) recurred within the 5-year follow-up period. Among 35 subtotally resected grade I meningiomas with MIB-1 values of ≤ 4%, only four (11%) recurred in five years. In the multivariable analysis, preoperative meningioma volume ((*p* = 0.02, HR 1.02) per 1 cm^3^ increase) and 4% cutoff for MIB-1 ((*p* = 0.02, HR 3.9) remained significant with respect to 5-year recurrence. Consistent with prior studies, the MIB-1 index of 4% seems to identify a subgroup of meningioma patients with low risk of recurrence. MIB-1 is likely to offer utility especially in designing individualized and less frequent imaging follow-up strategies after resection of WHO grade I meningiomas, where molecular genetics are not defined routinely.

Clinical trial number: not applicable.

**Supplementary Information:**

The online version contains supplementary material available at 10.1007/s10143-026-04345-3.

## Introduction

Meningiomas are the most common primary intracranial tumors in adults [1, 2]. Although generally benign in behavior, they demonstrate substantial variability in growth and recurrence patterns [3, 4]. Consequently, identifying reliable prognostic markers is essential for improving outcome prediction, guiding perioperative management and individualizing long-term postoperative follow-up strategies. While the World Health Organization (WHO) histological grading system remains the diagnostic standard, its prognostic accuracy is limited—particularly in predicting recurrence in WHO grade I meningiomas [5–7]. This has led to an increased interest in additional prognostic indicators, with Ki-67/MIB-1 gaining particular attention for its potential to predict recurrence [8–11]. Its widespread availability as part of routine pathological assessment further supports its usability as a prognostic biomarker.

Ki-67/MIB-1 measures tumor cell proliferation and has shown prognostic relevance across various cancers, including gliomas, where specific thresholds are already established [12–15]. In contrast, no universally accepted threshold values exist for meningiomas [16]. Suggested thresholds typically range from 3% to 5%, but these are often based on institutional practices rather than empirical evidence [17–19]. Recently, a retrospective study of 404 WHO grade I-III meningiomas operated between 1994 and 2015 in the US proposed a threshold of 4.1% for predicting around 10-year recurrence-free survival [20]. Notably, the study did not report a standard follow-up time for the study cohort.

The aim of this study was to validate and extend recent findings [20]. Based on the previous work, we hypothesized that an MIB-1 index threshold around 4% would predict meningioma recurrence within five years. We further evaluated whether prognostic performance improves when accounting for the extent of resection and presumed embryologic origin (i.e., neural crest vs. mesoderm). Prior studies indicate that presumed embryologic origin correlates with meningioma molecular profiles, including mitotic activity [21]. Establishing reliable MIB-1 thresholds below which recurrence is highly unlikely could enable more individualized, cost-effective and risk-adapted postoperative imaging strategies.

## Methods

### Study design and cohort

This retrospective cohort study included adult (≥ 18 years old) patients who underwent surgical resection for a histopathologically confirmed meningioma at Helsinki University Hospital between January 2005 and December 2018. Patient identification and data collection procedures have been previously described in detail [22]. Briefly, cases were identified using the International Classification of Diseases, 10th Revision (ICD-10) diagnostic code D32 (benign neoplasm of cerebral meninges). Clinical, surgical, radiological, and pathological data were extracted from institutional electronic health record systems, including Opera (GE Healthcare, Chicago, Illinois, United States), Uranus (CGI, Helsinki, Finland), RADU (L-Force, Helsinki, Finland), Qpati (Tietoevry, Espoo, Finland), and the hospital’s picture archiving and communication system.

## Inclusion criteria

Patients were included if they met all the following criteria: (1) adult patient (≥ 18 years old) who underwent surgical resection for histologically confirmed intracranial meningioma, (2) no prior surgical or radiation treatment for the observed meningioma, (3) immunohistochemistry results available for the Ki-67/MIB-1 index, and (4) the minimum of five years of clinical and radiological follow-up. In patients with multiple meningiomas, each surgically resected meningioma was treated as an independent case. Cases were analyzed as independent observations. All meningioma patients in the cohort had at least five years of follow-up, and outcomes are reported mainly as 5-year results for the entire cohort. Postoperative radiotherapy was not used as an exclusion criterion.

## MIB-1 index

The Ki-67/MIB-1 proliferation index had been analyzed as part of routine histopathological evaluation. In many cases, due to meningioma heterogeneity, the MIB-1 index was reported as a range (e.g., 10–12%). To standardize the data and facilitate quantitative analyses, each case was assigned a single representative MIB-1 index equal to the midpoint of the reported range. Unlike for WHO grade II and III meningiomas, however, genetic prognostic profiling was not constantly performed for who Grade I meningiomas.

## Outcome definitions

Patients were divided by the extent of resection, defined by the first postoperative MR imaging and surgeon’s operative report. In accordance with European association of neuro-oncology (EANO) guidelines, the extent of resection was confirmed with MRI and classified as either gross total resection (GTR) or subtotal resection (STR) [23]. GTR was defined as no residual tumor on postoperative MRI, whereas all other cases were considered STR. The study hospital has not used the Simpson grading scale systematically, partially due to presented criticism regarding its subjectivity and to its unsuitability in the setting of modern era meningioma surgery [24–26]. Generally Simpson Grade I, II and III resections can be considered GTR, and grade IV STR [27]. Meningioma recurrence was defined as radiological evidence of meningioma regrowth or progression of a residual meningioma on follow-up MR imaging after the first postoperative MRI. Reoperation was defined as a second tumor surgery due to the progression of the previously operated index meningioma. Surgeries performed for postoperative complications, such as infections and hematomas, were not classified as reoperations.

## Follow-up protocol

Per the hospital’s follow-up protocol, patients with WHO grade I meningiomas who underwent GTR received MRI scans at two and five years post-surgery. In contrast, patients with WHO grade II-III meningiomas or those who underwent STR were typically monitored annually with MRI. All cases included in this study underwent MRI at the five-year follow-up mark.

### Embryological origin

**Fig. 1:**
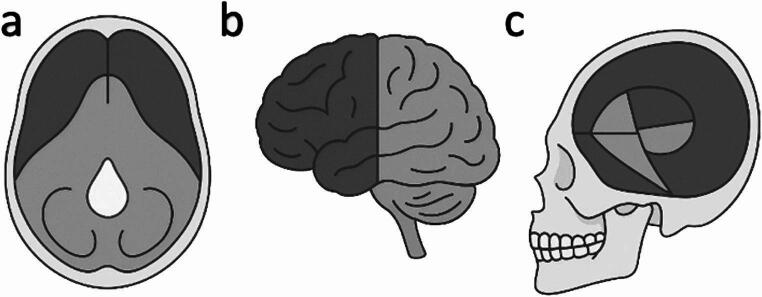
Schematic illustration of the spatial distribution of meningiomas by embryologic origin. Regions of neural crest origin are shown in dark gray, and regions of mesodermal origin in light gray. (**A**) Skull base. (**B**) Convexity and (**C**) Falx and tentorium. Image based on Boetto et al. [21]

Meningiomas were classified as either being neural crest or mesoderm derived, based on anatomical location. Meninges covering the cerebral convexity, falx cerebri and tentorium cerebelli derive predominantly from neural crest cells, while those at the skull base, posterior fossa and spine arise primarily from mesodermal origin [21]. The classification was based on the principles proposed by Boetto et al. [21] and is illustrated in Fig. 1. In borderline cases, classification was determined based on the predominately involved anatomical site.

### Statistical analysis

The added prognostic value of routine MIB-1 assessment is negligible in grade II-III meningiomas (they undergo comprehensive molecular genetic profiling), whereas it may provide meaningful additional information in grade I meningiomas. For this reason, coupled with the rarity of grade III meningiomas in our cohort (*n* = 13, 2.6%) of the whole cohort), we focused our analyses on grade I meningiomas and combined grades II and III into a single group. In line with the methodology used in the previous study [20], we conducted a receiver operating characteristic (ROC) curve analysis to evaluate the diagnostic accuracy of the MIB-1 labeling index in predicting two binary outcomes: meningioma recurrence and the need for reoperation. ROC curves were generated using the *pROC* package in R (version 4.3.1) [28], and the area under the curve (AUC) was calculated to quantify the discriminatory performance of the MIB-1 index for each outcome.

In instances with sufficient statistical discriminatory ability, defined as an AUC ≥ 0.70 [29], we further examined the distribution of MIB-1 values using boxplot analysis to determine threshold values, with outliers serving as candidates for clinically meaningful cutoff points. Boxplot analysis was used to detect anomalous data points (i.e., cutoff values where no recurrences are seen), and the independent prognostic value of the identified cutoff value was further tested in multivariable analysis. The objective of establishing an MIB-1 index threshold was to define a cutoff value below which meningioma recurrence is exceedingly unlike (i.e., a rule-out threshold), rather than to achieve the highest possible overall discriminatory accuracy, as would be prioritized in a standard ROC AUC analysis.

## Results

### Study cohort

We identified a total of 505 operated meningioma patients fulfilling the inclusion criteria. The general characteristics of the study cohort are highlighted in Table 1. Outcomes are highlighted in Table 2. During the five-year follow-up period, radiological recurrence was observed in 45/224 (20.1%) of grade I meningiomas after GTR and 21/58 (36%) after STR. For grade II-III meningiomas, the corresponding figures were 58/170 (34.1%) and 39/53 (74%).

**Table 1:** General characteristics of study patients

Number of patients,*n*	505
Sex, *n* (%)	
Woman	340 (67.3%)
Man	160 (31.7%)
Not identified	5 (1.0%)
Age, median (IQR)	59.0 (47.0–70.0)
Meningioma location, n (%)	
Neural Crest	274 (53.2%)
Anterior convexity	110 (40.1%)
Parasagittal	86 (31.4%)
Falx	50 (18.2%)
Tentorial	18 (6.6%)
Intraventricular	10 (3.6%)
Mesoderm	231 (46.8%)
Skull base	163 (70.6%)
Posterior fossa	54 (23.4%)
Posterior convexity	14 (6.1%)
WHO classification, n (%)	
Neural Crest	274 (53.2%)
I	127 (46.4%)
II	136 (49.6%)
III	11 (4.0%)
Mesoderm	241 (46.8%)
I	155 (64.3%)
II	74 (30.7%)
III	2 (0.8%)
Preoperative meningioma volume in cm^3^, median (IQR)	27.3 (9.2–53.7)
MIB, median (IQR)	6% (4%-10%)
Recurrence free follow-up in months,median (IQR)	60.0 (38.0–60.0)

*IQR*  Interquartile range, *WHO* World Health Organization

**Table 2:** Outcomes of study patients

Extent of resection, *n* (%)	
GTR	394 (78.0%)
I	224 (56.9%)
II	164 (41.6%)
III	6 (1.5%)
STR	111 (22.0%)
I	58 (52.3%)
II	46 (41.4%)
III	7 (6.3%)
Recurrence, *n* (%)	
No	342 (67.7%)
I	216 (63.2%)
II	126 (36.8%)
Yes	163 (32.3%)
I	66 (40.5%)
II	84 (51.5%)
III	13 (8.0%)
Reoperation, *n* (%)	
No	434 (85.9%)
I	255 (58.8%)
II	172 (39.6%)
III	7 (1.6%)
Yes	71 (14.1%)
I	29 (40.8%)
II	39 (54.9%)
III	6 (8.5%)

*GTR* gross total-resection, *STR* subtotal-resection

### MIB-1 Index and ROC AUC analyses

**Fig. 2:**
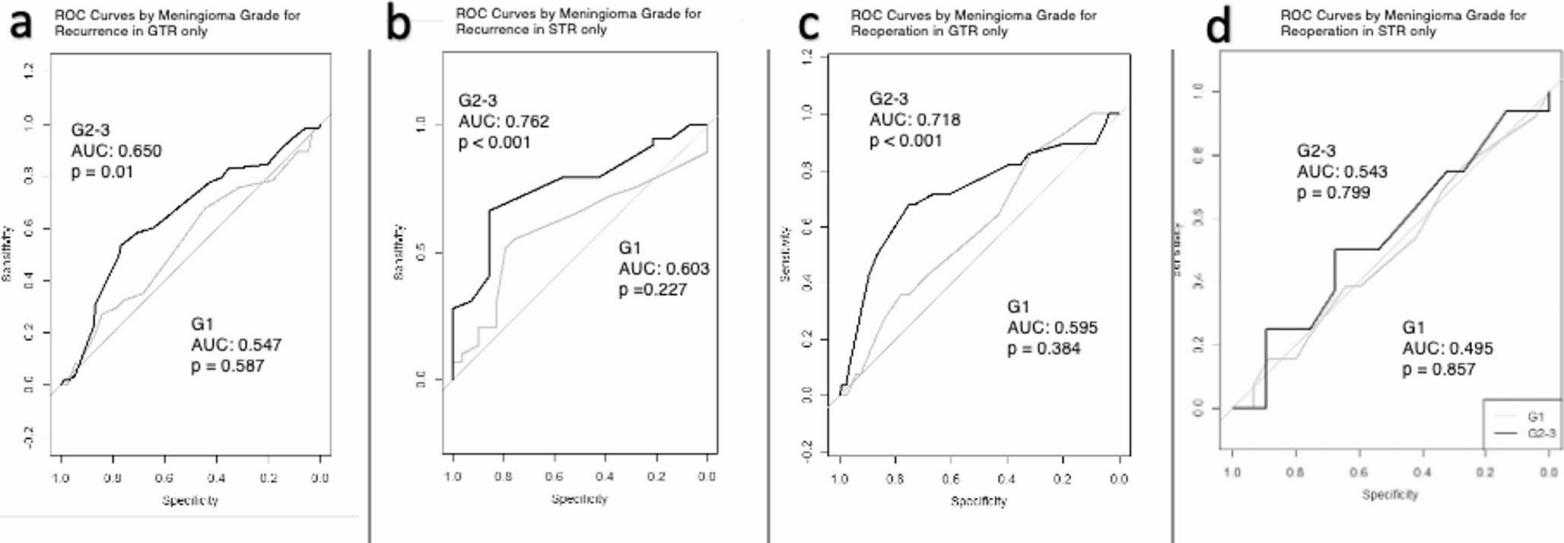
Receiver operating characteristic (ROC) curves illustrating the discriminatory performance of the MIB-1 proliferation index stratified by meningioma grade. (**A**) Prediction of meningioma recurrence after gross total resection (GTR). (**B**) Prediction of meningioma recurrence after subtotal resection (STR). (**C**) Prediction of need for reoperation after GTR. (**D**) Prediction of need for reoperation after STR

The MIB-1 proliferation index did not reach any significance (AUC ≥ 0.70) in predicting recurrence after GTR meningiomas or after STR in WHO grade I tumors (Fig. 2A–B). In contrast, it showed fair (AUC 0.76) discrimination in STR WHO grade II–III tumors (Fig. 2B).

For predicting reoperation, the MIB-1 index achieved fair discriminative performance (AUC 0.72) in the GTR WHO grade II–III subgroup (Fig. 2C), but not in STR cases regardless of grade (Fig. 2D).

### MIB-1 cutoff value and recurrence of WHO grade I meningiomas

In univariable Cox regression analysis of the entire cohort, variables significantly associated with 5-year recurrence included sex, preoperative meningioma volume, tumor location, WHO grade, presumed embryological origin, extent of resection, and 4% MIB index cutoff (Supplementary Table 1). Given that surveillance strategies for WHO grade II-III meningiomas are primarily guided by molecular characteristics rather than MIB-1 we focused subsequent analyses on subtotally resected WHO grade I meningiomas. In this subgroup, univariable Cox regression analysis identified preoperative tumor volume, location, WHO grade and the 4% MIB cutoff as associating variables with 5-year recurrence (Table 3), while in multivariable Cox regression analysis, preoperative tumor volume (*p* = 0.02, HR 1.0) and MIB > 4% (*p* = 0.02, HR 3.9) remained independently associated with 5-year recurrence (Table 3). There was no strong correlation (*p* = 0.51) between MIB ≥ 4% and preoperative meningioma volume in STR grade I meningiomas (the Wilcox-test). Among GTR WHO grade I meningiomas MIB > 4% was not associated with recurrence (*p* = 0.43, data not shown).

**Table 3:** Results of Cox proportional hazards regression analysis for 5-year recurrence among subtotally resected WHO grade I meningiomas. Significant (p<0.05) results are highlighted in bold

Variable	Count	Univariable *p*-value	Multivariable *p*-value and HR (95% CI)
Number of patients, *n*	58		
Sex, *n* (%)			
Woman	43 (74.1%)		
Man	13 (22.4%)	*p*=0.51	
Not identified	2 (3.4%)	*p*=0.81	
Age at operation, median (IQR)	58.0 (46.3-69.8)	*p*=0.08	
Preoperative meningioma volume in cm^3^, median (IQR)	25.2 (9.8-58.5)	***p*** **=0.01**	***p*** **=0.02, HR 1.02 (1.00-1.03) per 1 cm** ^**3**^ **increase**
Meningioma location, *n* (%)			
Convexity	4 (6.9%)		
Other	54 (93.1%)	*p*=1.00	
Embryological origin, *n* (%)			
Mesoderm	42 (72.4%)		
Neural Crest	16 (27.6%)	***p*** **=0.02**	p=0.24
MIB, *n* (%)			
≤4%	35 (60.3%)		
>4%	23 (39.7%)	***p=0.04***	**p=0.02, HR 3.9, (1.2-11.9)**

*IQR* Interquartile range, *WHO *World Health Organization, *GTR* gross total-resection, *STR* subtotal-resection, *HR* Hazard ratio, *CI* Confidence interval

Among the 58 STR WHO grade I meningiomas, the cumulative recurrence rate was 36% in five years. Four (7%) recurrences occurred within the first year, six additional (10%) recurrences occurred by two years and 11 additional (19%) recurrences had occurred within five years. Among the 35 STR WHO grade I meningiomas with MIB-1 ≤ 4%, no recurrences occurred within the first year, one recurrence (3%) occurred by two years, and three additional recurrences (9%) occurred within five years. Altogether, among 35 STR grade I meningioma patients with MIB ≤ 4%, the cumulative recurrence rate was 11% in five years. All these recurrences occurred in meningiomas located near the optic nerves.

Among the 224 GTR WHO grade I meningiomas, the cumulative recurrence rate was 20.1% in five years. Seven (3.1%) recurrences occurred within the first year, 19 additional (8.5%) recurrences occurred by two years, and 19 additional (8.5%) recurrences had occurred within five years. Among the 96 GTR WHO grade I meningiomas with MIB-1 ≤ 4% two recurrences (2%) occurred within the first year, two additional recurrences (2%) occurred by two years, and eight additional recurrences (8%) occurred within five years. Altogether, among 96 GTR grade I meningioma patients with MIB ≤ 4%, the cumulative recurrence rate was 13% in five years.

### MIB-1 cutoff values and WHO Grade II and III meningiomas

**Fig. 3:**
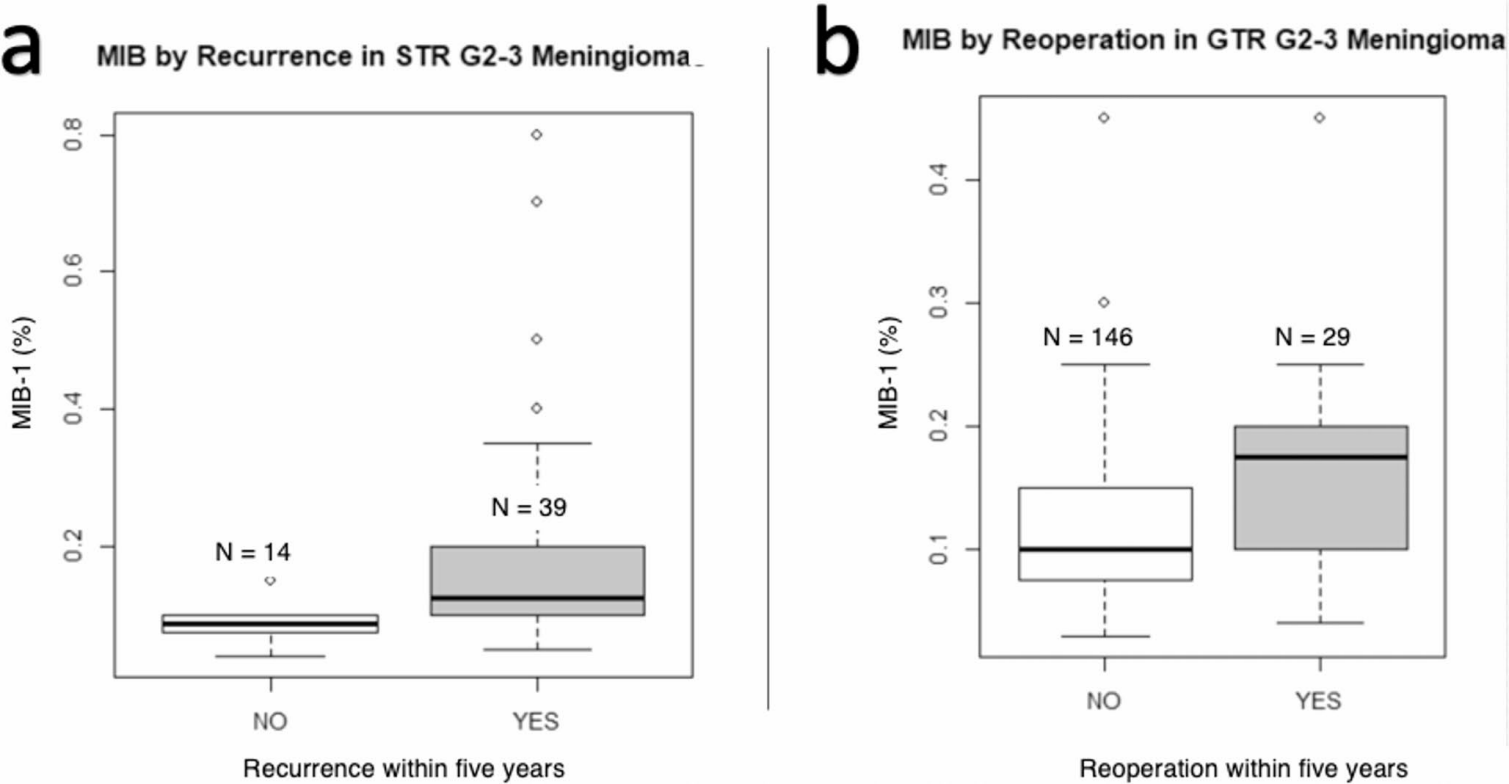
Boxplots showing MIB-1 proliferation index values for (**A**) subtotally resected (STR) GII-III meningiomas with recurrence during follow-up, and for (**B**) gross totally resected (GTR) GII-III meningiomas with reoperation during follow-up

Among 53 WHO grade II–III meningiomas treated with STR, a cut point of MIB-1 ≤ 5% was identified for a low recurrence risk (Fig. 3B). In more detail, five patients (9%) had MIB-1 ≤ 5% (all WHO grade II), and none of these experienced recurrence during the five-year-long follow-up.

In a cohort of 175 patients with WHO grade II–III meningiomas treated with GTR, a box-plot analysis identified an MIB-1 threshold of ≤ 4% (Fig. 3A). Of these 175 patients, 15 (8.6%) had an MIB-1 index ≤ 4% (all WHO grade II), and none required reoperation during a five-year-long follow-up.

### Embryological origin

In the subset of WHO grade I meningiomas treated with STR, neural crest origin associated with a higher five-year recurrence risk in univariable analysis (*p* = 0.02.) However, this association was no longer statistically significant in the multivariable analysis (*p* = 0.24, Table 3).

### Postoperative radiation

Adjuvant radiation was administered to 15 of 394 GTR patients (3.8%) and 19 of 111 STR patients (17.1%). MIB-1 indices ranged from 5% to 40% in patients receiving adjuvant radiation. None of the patients identified by our threshold values received radiation during the five-year follow-up.

## Discussion

There is a need for tools to improve the individualized long-term follow-up strategies of particularly subtotally resected grade I meningiomas. Our results suggest that MIB-1 proliferation index threshold of 4% enables the identification of operated grade I meningioma patients who may safely undergo less frequent MR follow-up imaging. Among the predictors of recurrence analyzed, MIB-1 ≥ 4% was independently associated with recurrence and represented one of the strongest predictors of recurrence in STR grade I meningiomas. Currently, MIB-1 assessment is not part of routine diagnostic practice in every center for WHO grade I meningiomas. However, based on our results, this inexpensive test, which is already performed routinely in other tumor types, could offer benefits by enabling more personalized MR follow-up strategies for operated WHO grade I meningiomas patients, instead of the current annual MR imaging surveillance. In higher grade meningiomas, comprehensive molecular profiling has become mandatory, and the role of MIB-1 as a sole prognostic marker is therefore limited.

Current European Association of Neuro-Oncology guidance recommends annual MRI for WHO grade I meningiomas and MRI at least every six months for WHO grade II-III meningiomas following treatment for the first five years [23]. Our study included 282 WHO grade I meningiomas, of which 58 (20.6%) belonged to the STR group. Among the STR group grade I meningiomas, 35 (60%) out of 58 had an MIB-1 index ≤ 4% and four (11%) radiologic recurrences were observed in this subgroup over the five-year period. In the GTR group meningiomas, 96 (42.9%) out of 224 had an MIB-1 index ≤ 4%, and 12 (12.5%) recurrences occurred within 5 years in this subgroup. Overall, in our cohort, 46.5% (131 out of 282) of all resected (both GTR and STR) grade I meningiomas had an MIB-1 index ≤ 4%. Given that grade 1 meningiomas account for approximately 80% of all resected meningiomas [2], these figures suggest that over a third of all resected meningiomas would be WHO grade I tumors, with MIB-1 ≤ 4%, which could potentially be followed much less frequently than annually. This would represent a significant change and resource savings compared to current surveillance recommendations.

We tried a novel approach combining an embryology-informed classification of meningiomas with MIB-1, to see if it offers potential for enhancing prognostic accuracy when integrated with molecular-biological markers. Combined with presumed embryologic origin, the MIB-1 proliferation index did not provide significant benefit in the assessment of recurrence risk for WHO grade I meningiomas.

No clinically relevant MIB-1 proliferation index threshold was identified for predicting recurrence following GTR of meningiomas. Among the 96 (42.9%) of GTR grade I meningiomas with MIB-1 index ≤ 4%, 12 (13%) recurred within 5 years. Of the 128 (57.1%) GTR grade I meningiomas with MIB-1 index > 4%, 33 (25.8%) recurred within 5 years. However, this difference did not reach statistical significance given the limited sample size of the subcohorts. Among STR WHO grade I meningiomas with an MIB-1 index ≤ 4%, all recurrences occurred in proximity to the optic nerve. Similarly, in GTR WHO grade I meningiomas with MIB-1 index ≤ 4%, recurrences occurred also in the proximity of the optic nerve in 36% of cases. These figures considered, it might be reasonable to continue standard imaging surveillance for WHO grade I meningiomas resected near the optic nerve, while adopting a considerably less frequent follow-up protocol for those located farther from the optic nerve with an MIB-1 index < 4%.

Prior research has investigated the utility of the MIB-1 proliferation index in predicting meningioma recurrence, with reported values below 3–5% associated with reduced recurrence risk [17–20]. These studies primarily focused on specific meningioma subtypes, examining either skull base [17, 18] or convexity [19] tumors.

The largest study to date (*n* = 401) and the first to employ data-driven methodologies, encompassed meningiomas from all anatomical locations and proposed a MIB-1 cutoff of 4.1% for predicting recurrence [20]. However, this study’s lack of stratification resulted in a suboptimal AUC value of less than 0.7, indicating poor predictive performance [24]. Furthermore, despite a substantial median follow-up of 129 months, the inclusion of patients with follow-up periods as short as three months raises concerns about potential follow-up bias. The author’s use of a Youden-index-derived cutoff value of 4.1%, while statistically optimized, may not directly translate to practical changes in follow-up imaging intervals.

Unlike prior studies, our aim was to evaluate whether MIB-1 could identify a subgroup of patients in whom les intensive follow-up might be considered. In our cohort, the proposed 4% MIB-1 cutoff performed well in identifying patients with low recurrence risk following STR of WHO grade I meningiomas. Among STR and GTR WHO grade I meningiomas with MIB ≤ 4%, the recurrence rate was only 11–13%, and al recurrence occurred often near the optic nerves. Meningiomas located near critical structures may show earlier progression, as even small growth becomes clinically symptomatic.

Given the large number of WHO grade 1 meningiomas and their generally benign nature, full molecular profiling is not feasible in routine clinical practice due to the cost involved. MIB-1, as a cost-effective immunohistochemical marker, could serve as a practical alternative for risk stratification in grade I meningiomas. This could enable more individualized surveillance strategies, potentially reducing imaging and alleviating patient anxiety related to repeat imaging. Establishing reliable cutoff points for non-recurrence (rather than solely for recurrence) is critical for clinical decision-making, as it enables confident de-escalation of follow-up in truly low-risk patients.

However, MIB-1 captures only mitotic activity and does not account for specific genetic alterations known to drive meningioma progression and recurrence [30]. Methylation-based classifications may provide more refined biological risk stratification [31–33]. Nevertheless, MIB-1 remains widely available, cost-effective, and reproducible in routine neuropathological practice, and it may serve as an alternate method to expensive molecular risk profiling in assessing recurrence risk particularly of patients with postoperative residual grade I meningiomas. While these findings are promising, they are insufficient to alter current clinical practice without prospective validation.

This retrospective, single-center study conducted at a tertiary care institution has several limitations. Although the cohort is the largest reported to date, stratification by extent of resection and WHO grade resulted in smaller subgroups, reducing statistical power for these analyses. Moreover, we did not use the Simpson grading to categorize the extent of resection as a predictor of meningioma recurrence, since the grading has not been employed by the vast majority of study neurosurgeons during the study period. This may complicate comparison with the results of studies that used the controversial grading introduced in 1957. The EANO stated in its latest recommendations for the diagnosis and treatment of meningiomas that the extent of resection is often defined as either GTR (i.e. no residual solid tumor) or STR. This definition has also been adopted by research organizations such as the European Organization for Research and Treatment of Cancer and the Radiation Therapy Oncology Group [23].

In cases where the MIB-1 proliferation index was reported as a range to account for intratumoral heterogeneity, we used the midpoint for statistical analyses. While this approach preserves biological variability and is arguably more representative than selecting a single, potentially non-representative value, it introduces some arbitrariness. Additionally, WHO grade II–III meningiomas were overrepresented due to selective MIB-1 assessment, which is not routinely performed for all WHO grade I meningiomas at our hospital, likely skewing the overall MIB-1 distribution upward. Moreover, recurrence was defined radiographically during follow-up, potentially introducing detection bias due to variations in imaging frequency or interpretation. However, adherence to a standardized institutional protocol, with five-year long follow-up imaging available for the cohort, likely mitigates this bias, as the endpoint was the presence or absence of recurrence within a fixed period rather than time to recurrence.

We did not assess whether radiographic recurrence was clinically meaningful, as our primary objective was to determine whether there exists a subpopulation that may not require frequent follow-up MR imaging. Another key limitation of this study is that postoperative tumor volume could not be meaningfully evaluated as a risk factor for recurrence. Immediate postoperative MRI within 72 h after surgery was not performed in the great majority of patients, precluding reliable assessment of the role of residual tumor volume. Finally, the single-center design of this study may limit the generalizability of our findings. However, we believe the results are sufficiently robust to serve as a hypothesis-generating basis for future studies. On a positive side, in our cohort, no patient with an MIB-1 value below the cutoff values was re-operated or received postoperative radiotherapy, minimizing potential confounding by postoperative treatments in the low-MIB-1 group.

## Conclusions

Consistent with prior studies, an MIB-1 index of 4 appears to identify a subgroup of meningioma patients with a low risk of recurrence. MIB-1 is unlikely to provide a substantial additional value in WHO grade II-III meningiomas, where molecular genetic analyses have become the gold standard for risk stratification. In contrast, MIB-1 may be particularly useful in guiding individualized imaging follow-up strategies after resection of WHO grade I meningiomas, where molecular profiling is not routinely performed and is often not feasible due to cost and the relatively limited additional clinical benefit.

## Supplementary Information

Below is the link to the electronic supplementary material.

Supplementary Material 1

## Data Availability

The datasets were processed exclusively within the institution’s secure cloud environment. Under applicable local data-privacy laws and institutional policy, individual-level data cannot be exported or shared outside this environment. Accordingly, the data are not publicly available and cannot be provided to third parties.
