# Correction to: MIB-1 index predicts low recurrence risk after subtotal resection of meningiomas: a retrospective study of 505 patients

**DOI:** 10.1007/s10143-026-04393-9

**Published:** 2026-07-11

**Authors:** Joonas Laajava, Jenni Määttä, Miikka Korja

**Affiliations:** Department of Neurosurgery, University of Helsinki and Helsinki University Hospital, P.O. Box 266, FI-00029 Helsinki, Finland

**Correction to: Neurosurgical Review**

10.1007/s10143-026-04345-3

The authors regret that the author names were captured incorrect as below in the original published article.

Laajava Joonas^1^ · Määttä Jenni^1^ · Korja Miikka^1^

The correct author names are shown below.

Joonas Laajava^1^ · Jenni Määttä^1^ · Miikka Korja^1^

The original article online has been corrected.

