# Supplementary material for: MIB-1 index predicts low recurrence risk after subtotal resection of meningiomas: a retrospective study of 505 patients
Source: Neurosurg Rev. 2026 Jun 11;49(1):444. doi: 10.1007/s10143-026-04345-3 (PMC13260087; doi:10.1007/s10143-026-04345-3)
Supplement: Supplementary file 1 — Supplementary Material 1 [file 10143_2026_4345_MOESM1_ESM.docx]

**Supplementary Table 1.** Results of Cox proportional hazards regression analysis for 5-year recurrence in the entire cohort. Significant (p<0.05) results are highlighted in bold.

| Variable | Count | Univariable p-value |
| --- | --- | --- |
| Number of patients, n | 505 |  |
| Sex, n (%)  Woman (R)  Man  Not identified | 340 (67.3%)  160 (31.7%)  5 (1.0%) | **p<0.01, HR 1.8 (1.3-2.4)**  p=0.63 |
| Age at operation, median (IQR) | 59.0 (47.0-70.0) | p=0.65 |
| Preoperative meningioma volume in cm^3^, median (IQR) | 27.3 (9.2-53.7) | **p=0.01, HR 1.01 (1.00-1.01) per 1 cm^3^ increase** |
| Meningioma location, n (%)  Convexity (R)  Skull Base  Parasagittal  Posterior fossa  Falx  Tentorial  Intraventricular | 124 (24.6%)  163 (32.3%)  86 (17.0%)  54 (10.7%)  50 (9.9%)  18 (3.6%)  10 (2.0%) | **p<0.01 HR 2.5 (1.6-3.9)**  **p<0.01 HR 2.7 (1.6-4.4)**  **p=0.03 HR 2.0 (1.1-3.6)**  **p=0.01 HR 2.2 (1.2-3.9)**  **p<0.01 HR 3.5 (1.7-7.6)**  p=0.99 |
| WHO classification, n (%)  I (R)  II-III | 282 (55.8%)  210 (44.2%) | **p<0.01 HR 2.1 (1.6-2.8)** |
| Presumed embryological origin, n (%)  Mesoderm (R)  Neural Crest | 241 (46.8%)  274 (53.2%) | **p=0.04 HR 1.4 (1.0-2.1)** |
| Extent of resection, n (%)  GTR (R)  STR | 394 (78.0%)  111 (22.0%) | **p<0.01 HR 3.1 (2.3-4.1)** |
| MIB, n (%)  ≤4% (R)  >4% | 140 (27.7%)  365 (72.3%) | **p<0.01 HR 1.8 (1.3-2.6)** |

IQR = Interquartile range, WHO = World Health Organization, GTR = gross total-resection, STR = subtotal-resection, HR = Hazard ratio, CI = Confidence interval. (R)=Reference category.
